# The L.C.C. and an Efficient Ambulance Service

**Published:** 1913-03-01

**Authors:** 


### The L.C.C. and an Efficient Ambulance Service.

*To the Editor of* The Hospital.

Sir,—As one who has attended practically all the meetings of the London County Council in the last three years, though not as a member of that body, I was particularly interested in your article on the ambulance question which appeared in The Hospital of February 22. Like you, I regret deeply that such a matter should ever have become even in the slightest degree a party question. You say that the blame for the present unsatisfactory condition of affairs rests upon both parties, but I am inclined to think that this does not give due credit to the Progressive party, who have at least made an effort during the three years to improve matters. Some of the Progressive members—and Mr. H. L. Jephson in particular—frequently endeavoured to raise the matter in the Council, but with little success, until the occasion in November last, when it was decided to apply to Parliament for power to co-ordinate the existing ambulance services in London. On that occasion Mr. Jephson moved an amendment requesting the General Purposes Committee to prepare a scheme for utilising such existing appliances as were obtainable, and for providing such further appliances as were necessary to make a complete and efficient scheme. That amendment, however, was defeated on a division, taken, I believe, on strict party lines, by 65 votes to 42.

I have heard it said that many Municipal Reformers would have been willing to support a more elaborate scheme if it had been brought forward. The only body which could bring up a larger scheme with any hope of success is the General Purposes Committee of the Council. Mr. Jephson's amendment would have led to the production of such a scheme, and it could have been carried if twelve members of the Municipal Reform party had voted against their leaders on this question.

I think this recitation of the facts will show that the Progressive party have at least made a sincere effort to carry out their election promises of three years ago.—Yours, etc.,

An Impartial Observer.

